# A pilot randomized controlled trial of a fruit and vegetable prescription program at a federally qualified health center in low income uncontrolled diabetics

**DOI:** 10.1016/j.pmedr.2021.101410

**Published:** 2021-05-31

**Authors:** Richard Bryce, Julia A WolfsonBryce, Alicia CohenBryce, Nicki Milgrom, Danny Garcia, Alicia Steele, Sean Yaphe, Denise Pike, Felix Valbuena, Lisa R. Miller-Matero

**Affiliations:** a Community Health and Social Services (CHASS) Center, 5635 W. Fort St., Detroit, MI 48209, USA; b Department of Family Medicine and Public Health Sciences, Wayne State University School of Medicine, 540 E. Canfield St., Detroit, MI 48201, USA; c Department of Family Medicine, Henry Ford Health System, 2799 W. Grand Blvd., Detroit, MI 48202, USA; d Department of Health Management and Policy, University of Michigan School of Public Health, Ann Arbor, MI, USA; e Providence VA Medical Center, Providence, RI, USA; f Department of Family Medicine, Alpert Medical School, Brown University and Department of Health Services, Policy, and Practice, Brown University School of Public Health, Providence, RI, USA; g Ecology Center, 339 E. Liberty St., Suite 300, Ann Arbor, MI 48104, USA; h Department of Behavioral Health, Henry Ford Health System, 2799 W. Grand Blvd., Detroit, MI 48202, USA

**Keywords:** BP, blood pressure (mmHg), BMI, body mass index, Fresh Rx, Fresh Prescription Program, FQHC, Federally Qualified Health Center, HbA_1_C, hemoglobin A1c concentration (percent, Fruit and vegetable prescription program, Low-income diabetics, Federally qualified health center

## Abstract

Eating a healthy diet is important for managing diabetes. Although there are high rates of diabetes in low-income urban areas, these patients often have limited access to fruits and vegetables. The 15-week Fresh Prescription (Fresh Rx) program was designed to improve access and consumption of fruits and vegetables among low-income patients with diabetes in Detroit, MI. The purpose of this study was to evaluate the effects of a fruit and vegetable prescription program on changes in hemoglobin A1C (HbA_1_C), blood pressure (BP), and body mass index (BMI) in patients with diabetes in a randomized controlled trial at a federally qualified health center (FQHC). Patients randomized to the Fresh Rx group (n = 56) were allotted up to $80 ($10 for up to eight weeks) for purchase of produce from a farmers market based at the FQHC. The control group (n = 56) received standard treatment plus information on community resources to improve health. Outcomes were compared at baseline and within three months of program completion. There were no significant between-group differences for any of the outcomes at program completion (p > .05); however, there was a small effect size for HbA1c (partial η^2^ = 0.02).

Within the Fresh Rx group, HbA1c significantly decreased from 9.64% to 9.14% (p = 0.006). However, no changes were noted within the control group (9.38 to 9.41%, p = 0.89). BMI and BP did not change from pre- to post-study in either group (p > .05). Results from this study offer preliminary evidence that produce prescription programs may reduce HbA_1_C in low-income patients with diabetes.

## Highlights

• Prevalence of diabetes in the United States continues to increase.
• The rate of increase of diabetes is higher among those living in poverty.
• Poor families are less likely to eat the recommended amount of fruits and vegetables.
• Fruit and vegetable prescription programs may help control type 2 diabetes.

## 1 Introduction

Diabetes is a serious chronic disease that affects the health of millions of people across the world. Unfortunately, the prevalence of diabetes continues to grow. The Global Burden of Disease report from 2015, showed that the prevalence of diabetes worldwide increased from 333 million persons in 2005 to 435 million persons in 2015 (GBD 2015 Disease and Injury Incidence and Prevalence Collaborators, 2016). Over that same time the prevalence of diabetes in the United States increased from 16.5 million persons in 2005 to 23.4 million persons in 2015 (Division of Diabetes Translation and Centers for Disease Control and Prevention, 2017). The rate of increase of diabetes is higher among racial and ethnic minorities (Ingelfinger and Jarcho, 2017) especially those living in poverty (Agardh et al., 2011). One reason for this disparity can be related to diet (Agardh et al., 2011). In 2018, at least 14.3 million American households were experiencing food insecurity (Agardh et al., 2011). Those experiencing food insecurity are unable to afford balanced meals and may cut back on the size of meals or go hungry because of too little money for food (Agardh et al., 2011). Households near or below the federal poverty line, and Black and Hispanic headed households are most affected by food insecurity (Coleman-Jensen et al., 2017). Low-income households are less likely to consume the recommended amount of fruits and vegetables (Grimm et al., 2012).

Increasing fruit and vegetable intake has been shown to decrease the risk of developing type 2 diabetes and is also beneficial in the treatment of type 2 diabetes (Ford and Mokdad, 2001). Fruit and vegetable prescription programs (sometimes called produce prescription programs) can be an effective way to encourage an increase in fruit and vegetable consumption in those living from poverty (Forbes et al., 2019, Freedman et al., 2013, Marcinkevage et al., 2019, Omar et al., 2017, Trapl et al., 2018, Weinstein et al., 2014). This incentive model typically allows a health care provider to “prescribe” fresh fruit and vegetables to patients experiencing diet-related chronic diseases while receiving nutrition education in a clinical setting. Fruit and vegetable prescription programs have been shown to encourage healthy eating habits (Aiyer et al., 2019, Cavanagh et al., 2017, Forbes et al., 2019, Freedman et al., 2013, Marcinkevage et al., 2019, Omar et al., 2017, Richie, 2019, Ridberg et al., 2019, Saxe-Custack et al., 2019, Snailer, 2019, Trapl et al., 2018, Weinstein et al., 2014, York et al., 2020), decrease the prevalence of food insecurity (Aiyer et al., 2019), and have been associated with an increase in fruit and vegetable consumption in both adults (Forbes et al., 2019, Freedman et al., 2013, Marcinkevage et al., 2019, Omar et al., 2017, Trapl et al., 2018, Weinstein et al., 2014) and children (Ridberg et al., 2019, Saxe-Custack et al., 2019). The 2018 Farm Bill authorized the Gus Schumacher Nutrition Incentive Program (GusNIP), which provides funding opportunities to conduct and evaluate fruit and vegetable prescription programs by low-income consumers.

Limited research to date has examined clinical outcomes related to fruit and vegetable prescription programs; all have been single group, pre/post-program analyses (Forbes et al., 2019, Freedman et al., 2013, Marcinkevage et al., 2019, Omar et al., 2017, Trapl et al., 2018, Weinstein et al., 2014). These studies have demonstrated a significant decrease in body mass index (BMI) (Cavanagh et al., 2017) and blood pressure (BP) (York et al., 2020), as well as improvement in blood glucose control (Richie, 2019, Snailer, 2019). A 2015 study of patients with type 2 diabetes participating in a fruit and vegetable prescription program found participants experienced a significant decrease in hemoglobin A_1_C percentage (HbA_1_C) (i.e., 9.54 to 8.83) (Bryce et al., 2017). However, weight and BP did not change from pre- to post-study (p > .05) (Bryce et al., 2017). Although these results are encouraging, more rigorous investigation (i.e., inclusion of a comparison group and randomization of participants) is needed to strengthen the degree of evidence of the impact of fruit and vegetable prescription programs.

To fill this gap, the goal of this study was to complete a pilot randomized controlled trial of patients with type 2 diabetes participating in a fruit and vegetable prescription program. We assessed changes in HbA_1_C, BP, and BMI to discern the impact on those that participated in a fruit and vegetable prescription program compared to those that received non-incentivized diabetes standard of care.

## 2 Methods

### 2.1 Program

The Fresh Prescription (Fresh Rx) Program is a fruit and vegetable prescription program that brings together the healthcare system and the food system. This fosters innovative relationships to enhance the understanding of the correlation between food choices and health, increase consumption of locally grown fruits and vegetables, and build a healthy sustainable food system. This promising approach to a healthier food system connects patients to fresh, locally grown produce while providing direct economic benefits to small and midsize farmers and improving health and quality of life for participants.

We conducted a pilot randomized controlled trial from June 1, 2018 through January 1, 2019 to test the effects of the Fresh Rx program on patients from a Federally Qualified Health Center (FQHC) in Detroit, MI. The majority of patients from this FQHC are of lower socioeconomic status, which reflects a typical urban FQHC in the United States (National Association of Community Health Centers, 2020). The design of this trial used the principals of community based participatory research to guide this investigation (Israel et al., 2010, Israel et al., 2005). A community advisory board was created with a mixture of academic and community partners to best evaluate the program and research process. This study was approved by the Institutional Review Board of the Henry Ford Health System.

The Fresh Rx program allotted up to $80 ($10 per visit for up to 8 visits) for purchase of fresh fruits and vegetables at that FQHC’s farmers’ market (referred to as the Mercado). The Mercado, which is located outside the entrance to the health center, is a collection of several local produce farmers. The Mercado operated every Thursday (9 am to 1 pm) and occurred over 15 weeks from June 2018 to October 2018. In addition to selling fresh produce, the Mercado also offers many other positive health promoting activities including cooking demonstrations, nutrition education and exercise events.

Fresh Rx participants were able fill their prescription at the Mercado for fresh produce up to 8 times during the 15-week Fresh Rx program. The visits could but did not necessarily need to be in consecutive weeks. The provided debit cards were loaded with the $10 stipend at each visit. The participants, as well as farmers at the Mercado, were educated about the program and signage at vendor booths reinforced eligible purchases, which included only fresh produce. Prepared foods and juices, even if they were fruit or vegetable based, were not an eligible purchase. At each market session, cooking demonstrations took place that reinforced healthy food options and how to prepare foods that were available at the Mercado. Participants could return to the Mercado at other times after the completion of their 8 visits but did not receive any further financial incentives.

### 2.2 Study participants

A list was generated from the electronic medical records of the FQHC, of all non-pregnant patients with type 2 diabetes who had a HbA_1_C > 8.0% over the 6 months prior to the start of the Fresh Rx Program (N = 530). Using simple randomization, the list was randomized into two groups: the intervention group and the control group. (n = 265 each group). The randomization occurred prior to enrollment to decrease the work burden of program coordination for both the Fresh Rx program and the subsequent research. Those selected for the intervention group and the control group were contacted by telephone and offered participation by a community health worker (CHW). Both the CHWs and potential participants were aware of which group they were randomized (intervention vs. control) prior to participation. Once agreeable to participate, both groups were brought into the center to sign an informed consent. At that time, they had their BP, weight and HbA_1_C measured.

All Fresh Rx participants completed a basic program orientation that included receiving their Fresh Rx debit card that could be used with Mercado vendors. All participants then had their BP, weight and HbA_1_C checked inside the FQHC after their last visit to the Mercado or within 3 months of the completion of the Fresh Rx program (January 1, 2019). The 3-month time period for follow up was chosen understanding the HbA_1_C test shows the average amount of glucose attached to hemoglobin has been over the previous 3 months (MedlinePlus, 2020).

All control group participants were given flyers describing all the health and wellness programs at the FQHC including the Mercado. This information is the standard of care that is shared with all of the patients with type 2 diabetes at the Detroit based FQHC. No incentive was given for the Mercado. Control group participants were then given a $10 gift card to a national brand pharmacy (no fresh produce available). The enrollment time for the control group overlapped the Fresh Rx group (June to September 2018). After a 3-month time period, control group participants returned and had a repeat BP, weight and HbA_1_C measured. They then were given a $20 gift card to the same national brand pharmacy.

### 2.3 Statistical analyses

Descriptive analyses were conducted to determine the percentages, means, and standard deviations of participant demographics and the number of times participants utilized the market. Chi-squared analyses and independent samples t-tests were conducted to determine whether there were any demographic or biometric data differences between the intervention and control groups at baseline. Analysis of covariances (ANCOVA) were also conducted to examine whether there were significant differences in HbA_1_C, BMI, and systolic and diastolic blood pressure readings between groups, controlling for baseline levels. Paired sample t-tests were conducted from pre- to post-program to evaluate changes in HbA_1_C, BMI, and systolic and diastolic blood pressure readings within the intervention and control groups. We also ran analyses using an intent-to-treat approach, such that we used baseline biometric data as the follow-up numbers for those who were lost to follow-up. Using this approach, we had similar results, and so we chose to present the data from those who completed the follow-up for ease of interpretation. All analyses were performed using SPSS version 25 (IBM, Armonk, NY) and statistical significance was considered at p < .05. Partial eta effect sizes were reported for ANCOVA analyses, with interpretation as 0.01, 0.09, and 0.25 being small, medium, and large, respectively. Cohen’s d effect sizes were reported for paired samples t-tests and included the correlation of the pre- and post-intervention variables in the calculation. Interpretation was small (0.2), medium (0.5), or large (0.8).

## 3 Results

**Fig. 1:**
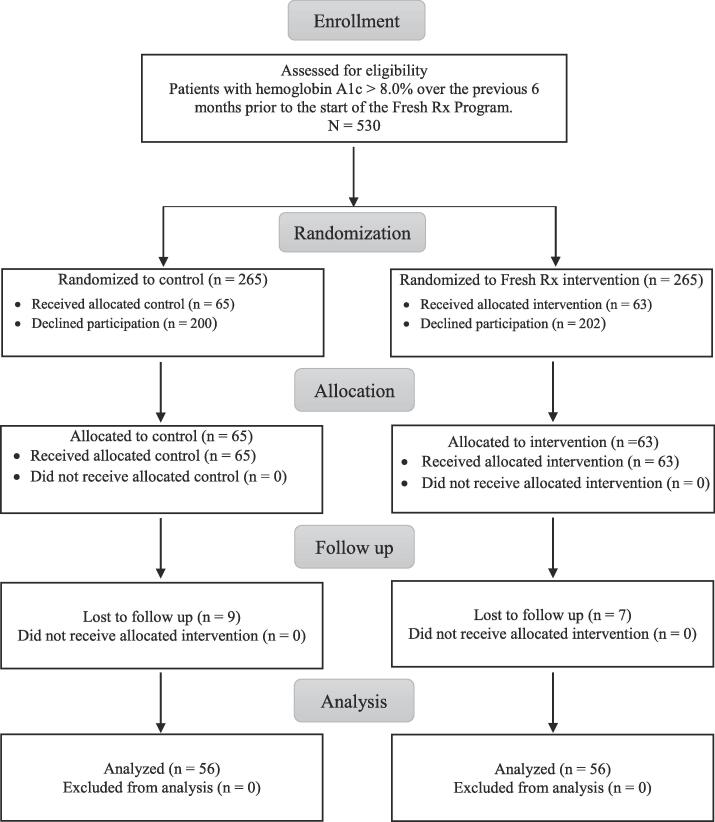
Flow of Participants through the Study.

Of the 265 adult, non-pregnant, patients with type 2 diabetes who were randomized to the intervention program and the control group, 23.8% (n = 63) and 24.5% (n = 65) agreed to participate, respectively (Fig. 1). There were 56 participants in the control group and 56 participants in the intervention group who had both baseline and follow-up data and were included in the final analyses. Characteristics of study participants are in Table 1. For both the intervention and control group, most participants were female, Latinx, and were either uninsured or underinsured. There were no significant differences between intervention and control groups for demographics or baseline HbA1c, BMI, or BP (Table 1). More than half of intervention participants had at least 5 market visits (58.9%, n = 33) during the 15-week program, and more than a quarter went to the Mercado at least 8 times and used all 8 prescriptions (28.6%, n = 16) (Table 2).

**Table 1:** Patient Demographics.

	Fresh Rx n = 56		Control n = 56			
Characteristics	n	%	n	%	X^2^	p
*Gender*						
Female	41	73.2	33	58.9	2.55	0.11
Male	15	26.8	23	41.1		

*Race/Ethnicity*						
Hispanic or Latino	41	73.2	37	66.1	1.27	0.53
Black/African American	12	21.4	17	30.4		
White/Caucasian	3	5.3	2	3.6		

*Insurance status*						
No insurance	23	41.1	20	35.7	1.54	0.46
Medicaid/Medicare	25	44.6	31	55.4		
Commercial insurance	8	14.3	5	8.9		

	M	SD	M	SD	t	p

Age, years	54.2	10.5	53.4	11.9	−0.37	0.71
Baseline HbA_1_C	9.69	2.10	9.38	1.99	−0.81	0.42
Baseline BMI	32.98	6.67	34.49	8.14	1.07	0.26
Baseline SBP	131.11	17.57	132.32	17.61	0.37	0.72
Baseline DBP	78.98	8.88	79.02	9.20	0.02	0.98

BMI, body mass index; DBP, diastolic blood pressure; ES, Effect Size; HbA_1_C, hemoglobin A1C percentage; M, Mean; SBP, systolic blood pressure.

**Table 2:** Number of visits to Mercado throughout the 15-week Fresh Rx program (n = 56).

Number of Visits	n	%
8	16	28.6
7	5	8.9
6	5	8.9
5	7	12.5
4	4	7.1
3	3	5.4
2	4	7.1
1	12	21.4

There were no significant differences between the control and intervention groups for any of the outcome variables (i.e., BMI, BP, or HbA1c) following the intervention; however, there was a small effect size for HbA1c (Table 3).

**Table 3:** Weight, Blood Pressure and Hemoglobin A_1_C.

	Fresh Rx Intervention Group (n = 56)					Control Group (n = 56)							
	Pre-Intervention M (SD)	Post-Intervention M (SD)	t^a^	p^a^	ES^b^	Pre-Intervention M (SD)	Post-Intervention M (SD)	t^a^	p^a^	ES^b^	F^c^	p^c^	ES^d^
BMI	32.98 (6.67)	33.26 (6.68)	−0.75	0.46	0.10	34.39 (8.14)	34.51 (8.05)	−0.11	0.91	0.11	0.24	0.63	0.002
Systolic BP (mm Hg)	131.11 (17.57)	130.21 (17.53)	0.42	0.68	0.06	132.32 (17.61)	134.00 (19.17)	−0.80	0.43	0.11	1.19	0.28	0.01
Diastolic BP (mm Hg)	78.98 (8.88)	78.23 (8.02)	0.72	0.47	0.10	79.02 (9.20)	78.32 (8.33)	0.67	0.51	0.09	0.003	0.96	<0.001
HbA_1_C (%)	9.69 (2.10)	9.15 (1.78)	2.86	0.006	0.38	9.38 (1.99)	9.41 (1.95)	−0.14	0.89	0.02	2.63	0.11	0.02

BMI, body mass index; BP, blood pressure; ES, Effect Size; HbA_1_C, hemoglobin A1C; M, Mean; SD, Standard Deviation.

a Within-group paired samples *t*-test.

b Cohen’s d effect sizes.

c Between-group ANCOVA, controlling for baseline levels.

d Partial eta squared effect sizes.

**Fig. 2:**
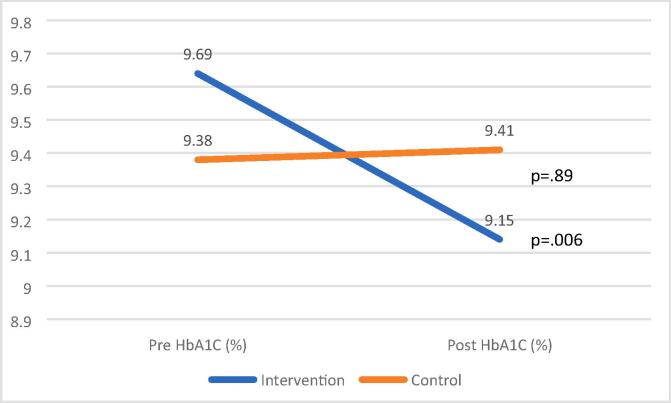
Hemoglobin A_1_C (HbA1C) Pre- and Post-Fresh Rx Intervention.

Because this was a pilot trial, we conducted within group analyses (i.e., intervention and control groups) to determine whether there was a signal to indicate a potential change from pre- to post-intervention (Table 3). Within the Fresh Rx group, HbA_1_C significantly decreased, with a small to medium effect size, while no changes were noted within the control group (Fig. 2 and Table 3). BMI and BP did not change from pre- to post-study in either group (p > .05).

## 4 Discussion

The findings from this pilot randomized control trial of a fruit and vegetable prescription program in a FQHC, suggest that such a program may assist in the management of HbA_1_C among patients with type 2 diabetes. This supports previous research that suggested this type of program may be useful for patients with type 2 diabetes (Bryce et al., 2017) and demonstrates that findings hold when including a comparison.

Fresh prescription programs may be effective as they give access of fresh produce to those that may have limitations due to food insecurity or live in food deserts (Hennessee, 2020). In more impoverished areas, many have limited ability to purchase fruits and vegetables due to cost or limited availability in stores selling fresh produce in their neighborhoods. These challenges can often lead to a poor diet (Swartz, 2018). When incentivizing people to eat more fruits and vegetables, they may be more likely to eat less junk food and consume healthier food. Also, they encourage participants to make the connection between nutrition and health (Boone-Heinonen et al., 2011). Thus, it is possible that the benefits could extend beyond the fruit and vegetable prescription program; however, future research is needed to examine longer term outcomes. Results from the evaluations of fruit and vegetable prescription programs have shown that participants consume more fruits and vegetables (Forbes et al., 2019, Freedman et al., 2013, Marcinkevage et al., 2019, Omar et al., 2017, Trapl et al., 2018, Weinstein et al., 2014). They also have demonstrated improved health outcomes in weight, hypertension and diabetes (Cavanagh et al., 2017, Richie, 2019, Snailer, 2019, York et al., 2020). The results of this pilot randomized controlled trial strengthen the evidence of the positive impact of fruit and vegetable prescription programs on FQHC patients with type 2 diabetes.

We did not see a change in BMI or BP in either the intervention group or the control group. From our data, it is unclear if weight and blood pressure are likely to respond positively to fruit and vegetable prescription programs. This has been shown in other studies (Ford and Mokdad, 2001) and although patient demographics and study time lengths are similar, all the studies including this one, have limited sample sizes. Also, we chose a 3-month follow-up window for participants as that corresponds best for the possible influence on the HbA_1_C test (MedlinePlus, 2020). The follow-up timeline of our study may have contributed to the lack of change in BMI and BP.

There are several strengths of this study that add to the current literature on fruit and vegetable prescription programs, including having a control group and having high retention rates across the intervention and control groups. Despite this, there are also some limitations that should be noted. First, the smaller than anticipated sample size likely prevented us from finding a significant between-group effect, given that the effect size was small. Increasing the sample size in a fully powered randomized control trial will allow us to better understand the significance of the pre- and post-biometric data. Second, randomization was conducted before entry into the study. This could have introduced bias and affected the statistical equivalence as CHWs and participants were aware of to which group they were randomized. Future research should conduct randomization into conditions after enrollment in the study.

One challenge we saw in the Fresh Rx program is many of those offered participation elected not to participate. Further, of those that were in the Fresh Rx group, there were varying levels of Mercado visits, with approximately 20% only attending once. This suggests that there may be barriers to participation in this program. Future research should evaluate barriers to implementation and methods in which to improve interest in participation, which could include increasing the hours of the market, expanding the length of the market season, expanding the stores/locations at which the benefit can be spent, and delivering the fresh produce to those that have challenges with transportation. It also would be important to examine fruit and vegetable prescription programs on different patient populations including those from more suburban or rural areas, those that reflect different races and ethnicities, as well as those with different socioeconomic status.

## 5 Conclusion

The findings of this study demonstrate the potential impact of fruit and vegetable prescription programs on the health of patients with type 2 diabetes. Future research should evaluate this type of program in a fully powered randomized controlled trial to examine efficacy and potential mechanisms of change. If these programs are found to be efficacious for improving HbA_1_C among patients with type 2 diabetes and low income/socioeconomic status, insurance companies may want to consider implementing routine fruit and vegetable prescriptions as a part of health care plans to improve patient health and the economic impact of type 2 diabetes.

## Funding

This work was supported by Blue Cross Blue Shield Foundation of Michigan, [grant number 002461.MG]

## CRediT authorship contribution statement

**Richard Bryce:** Conceptualization, Methodology, Writing - review & editing. **Julia A WolfsonBryce:** Conceptualization, Methodology, Writing - review & editing. **Alicia CohenBryce:** Conceptualization, Methodology, Writing - review & editing. **Nicki Milgrom:** Conceptualization, Methodology. **Danny Garcia:** Writing - review & editing. **Alicia Steele:** Writing - review & editing. **Sean Yaphe:** Writing - review & editing. **Denise Pike:** Conceptualization, Methodology, Funding acquisition. **Felix Valbuena:** Resources. **Lisa R. Miller-Matero:** Conceptualization, Methodology, Formal analysis, Validation.

## Declaration of Competing Interest

The authors declare that they have no known competing financial interests or personal relationships that could have appeared to influence the work reported in this paper.
